# Mechanical dyssynchrony alters left ventricular flow energetics in failing hearts with LBBB: a 4D flow CMR pilot study

**DOI:** 10.1007/s10554-017-1261-5

**Published:** 2017-11-02

**Authors:** Jakub Zajac, Jonatan Eriksson, Urban Alehagen, Tino Ebbers, Ann F. Bolger, Carl-Johan Carlhäll

**Affiliations:** 1 Division of Cardiovascular Medicine, Department of Medical and Health Sciences, Linköping University, 581 85 Linköping, Sweden; 2 Center for Medical Image Science and Visualization (CMIV), Linköping University, Linköping, Sweden; 3 Department of Clinical Physiology, Department of Medical and Health Sciences, Linköping University, Linköping, Sweden; 4 Department of Cardiology, Department of Medical and Health Sciences, Linköping University, Linköping, Sweden; 5 Department of Medicine, University of California San Francisco, San Francisco, CA USA

**Keywords:** Heart failure, Left bundle branch block, Left ventricular mechanical dyssynchrony, 4D flow CMR

## Abstract

The impact of left bundle branch block (LBBB) related mechanical dyssynchrony on left ventricular (LV) diastolic function remains unclear. 4D flow cardiovascular magnetic resonance (CMR) has provided reliable markers of LV dysfunction: reduced volume and kinetic energy (KE) of the portion of LV inflow which passes directly to outflow (*Direct Flow*) has been demonstrated in failing hearts compared to normal hearts. We sought to investigate the impact of mechanical dyssynchrony on diastolic function by comparing 4D flow in myopathic LVs with and without LBBB. CMR data were acquired at 3 T in 22 heart failure patients; 11 with LBBB and 11 without LBBB matched according to several demographic and clinical parameters. An established 4D flow analysis method was used to separate the LV end-diastolic (ED) volume into functional flow components based on the blood’s timing and route through the heart cavities. While the *Direct Flow* volume was not different between the groups, the KE possessed at ED was lower in LBBB patients (P = 0.018). *Direct Flow* entering the LV during early diastolic filling possessed less KE at ED in LBBB patients compared to non-LBBB patients, whereas no intergroup difference was observed during late filling. Pre-systolic KE of LV *Direct Flow* was reduced in patients with LBBB compared to matched patients with normal conduction. These intriguing findings propose that 4D flow specific measures can serve as markers of LV mechanical dyssynchrony in heart failure patients, and could possibly be investigated as predictors of response to cardiac resynchronization therapy.

**Electronic supplementary material:**

The online version of this article (doi:10.1007/s10554-017-1261-5) contains supplementary material, which is available to authorized users.

## Background

In the normal cardiac conduction system, the electric potential reaches the left ventricular (LV) myocardial walls nearly simultaneously, which enables the LV to contract and relax in a synchronous manner. Malfunction of the conduction system can lead to asynchronous LV pumping. In approximately one-fourth of cases heart failure is associated with left bundle branch block (LBBB) [1]. This electrical block in the main fascicle of the LV conduction system leads to dyssynchronous LV contraction and relaxation. Left ventricular dyssynchrony has been associated with increased morbidity and mortality, and prolongation of QRS duration has been identified as predictor of adverse events [2–4]. The LBBB-related mechanical dyssynchrony can contribute to LV diastolic dysfunction which in turn may augment the development of adverse cardiac remodeling [5].

LV mechanical dyssynchrony can be treated with cardiac resynchronization therapy (CRT) [6]. However, this therapy is expensive and the number of non-responders remains significant [7]. Reliable functional markers of dyssynchronous LV pumping that can predict response to CRT have proved elusive. In particular, little is known about the effects of LBBB on LV diastolic function which may contribute to therapeutic failures. Hence, there is a need for improved assessment of LV mechanical dyssynchrony for better identification of likely responders to CRT before device implantation.

Assessment of intracardiac blood flow demonstrates fundamental aspects of cardiac function in health and disease [8–11]. 4D flow cardiovascular magnetic resonance (CMR) is a versatile and promising method for the assessment of intracardiac flow [12–17]. LV flow-specific measures have recently emerged as markers of LV function: in normal hearts, the volume and energetics of the portion of LV inflow that passes directly to outflow, the *Direct Flow* component, reflect aspects of efficient LV systolic ejection [18]. Reduced volume and pre-systolic kinetic energy (KE) of the *Direct Flow* has been demonstrated in myopathic LVs compared to normal LVs [19, 20].

The vast majority of all studies of LV mechanical dyssynchrony focus on wall motion properties rather than aspects of intraventricular flow [21–24]. A consequence of LBBB is that the LV systolic contraction and early diastolic relaxation of the LV lateral wall will be delayed compared to especially the septum [25, 26]. A delayed outward motion of the LV lateral wall at early filling may influence the diastolic flow path and energetics of the inflowing blood. Therefore, we hypothesized that the volume and pre-systolic KE of *Direct Flow* would be further reduced in heart failure patients with myopathic LVs and LBBB compared to similarly dysfunctional and remodeled LVs without LBBB and that the disordered repolarization would mainly affect the *Direct Flow* entering during the early diastolic phase.

## Methods

### Study population

Twenty-two heart failure patients were enrolled; eleven patients with LBBB and eleven patients without LBBB matched according to LV ejection fraction (EF), LV end-diastolic volume (EDV) index, heart rate, age and gender. In both groups, etiology of heart failure was idiopathic dilated cardiomyopathy (from hereon referred to as nonischemic cardiomyopathy, NICM) in seven, and ischemic cardiomyopathy (ICM) in four. The patients were retrospectively included from a larger study group of heart failure patients enrolled at the Department of Cardiology, Linköping University Hospital.

Inclusion criteria for (1) ICM patients: diagnosed with ischemic heart disease based on pathological myocardial perfusion scintigraphy with typical ischemic appearance and/or MRI evidence of myocardial infarction using late gadolinium enhancement; at least mild systolic LV dysfunction (ejection fraction < 50%); (2) NICM patients: absence of other (secondary) etiology of dilated cardiomyopathy besides idiopathic; at least mild systolic LV dysfunction (ejection fraction < 50%); at least mild LV dilatation and; (3) LBBB patients: QRS duration > 120 ms and typical LBBB changes to the QRS complex and T wave. Exclusion criteria for all patients were contraindication for MRI examination, significantly irregular ventricular rhythm, heart rate < 40 bpm or > 100 bpm, more than mild to moderate valvular disease.

The study was approved by the Regional Ethical Review Board in Linköping and all subjects gave written informed consent before participation.

### Data acquisition and post processing

Time-resolved, three-directional, three-dimensional velocity data and morphological two-, three- and four-chamber long-axis and a stack of short axis images were acquired in all subjects using a clinical 3T MRI scanner (Philips Ingenia; Philips Medical Systems, Best, the Netherlands). The morphological images used for segmentation of ventricular volumes were acquired during end-expiratory breath-holds, using balanced steady-state free precession (bSSFP) imaging, and reconstructed into 30 timeframes. The number of slices varied according to the size of each patient’s heart. The short- and long-axis images had a resolution of 1.0 × 1.0 mm^2^ and a slice thickness of 8.0 mm. Other imaging parameters were: repetition time (TR), 2.8 ms; echo time (TE), 1.4 ms; flip angle, 45°; and parallel imaging with sensitivity encoding (SENSE) with a speed-up factor of 2–3.

**Fig. 1:**
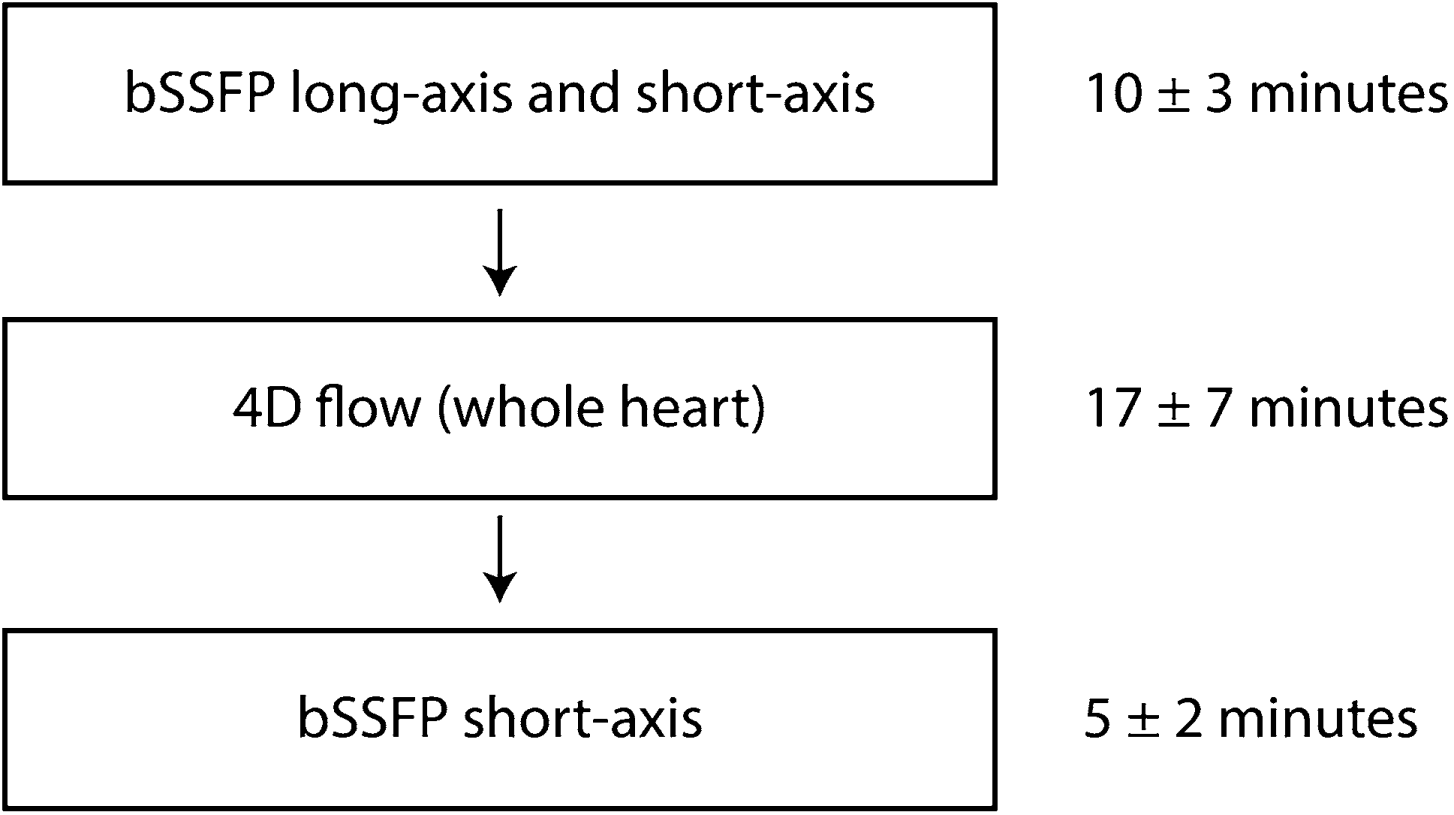
The CMR protocol and approximate scan times (mean ± SD). *bSSFP* balanced steady-state free precession

The velocity (4D flow) data were acquired in a volume encompassing the heart, using a gradient echo sequence with interleaved bipolar flow encoding gradients during free breathing, using retrospective navigator gating. Acquisition parameters were: velocity encoding (VENC), 120 cm/s; spatial resolution, 2.8 mm isotropic; flip angle, 10° (5° in subjects where contrast agent was not administered); TR, 4.4 ms, TE, 2.6 ms. A k-space segmentation factor of 3, parallel imaging with SENSE with a speed-up factor of 3 and elliptical k-space acquisition was applied. These settings gave a temporal resolution of 52.8 ms. All acquisitions were performed with the patients in supine position. The patients were given a gadolinium contrast agent (Magnevist, Bayer Schering Pharma AG) prior to the data acquisition for a late gadolinium enhancement study. The CMR protocol and approximate mean scan times are presented in Fig. 1.

After data acquisition the 4D flow data were reconstructed into 40 time frames on the scanner. The data were then post processed and analyzed on an offline station, using in-house developed software written in Matlab (The Mathworks Inc., Natick, Massachusetts, USA). The post processing procedure included correction for background errors by the use of a 4th order polynomial fitted to the static tissue in the thorax and phase wraps by the use of a temporal algorithm [27].

All subjects underwent standard electrocardiographic (ECG) and echocardiographic examinations adjacent in time to the MRI scan. The electrocardiographic examination was performed using a GE MAC 5500 HD by experienced nurses at the Department of Cardiology, Linköping University Hospital. An experienced investigator assessed the presence of LBBB according to standard definitions: QRS duration > 120 ms and typical LBBB changes to the QRS complex and T wave [28]. A conventional transthoracic echocardiographic examination using a Vivid E9 scanner (GE, Vingmed Ultrasound, Horten, Norway) was performed by experienced ultrasound technicians at the Department of Clinical Physiology, Linköping University Hospital. According to standard recommendations, an experienced investigator assessed the: presence of left sided regurgitant or stenotic valvular disease and LV diastolic function according to mitral and pulmonary venous inflow and mitral annular velocities in all patients as well as regional LV myocardial function in patients with ICM [29, 30].

### Data analysis

4D flow CMR measures have previously been evaluated compared to 2D phase-contrast CMR measures [12]. A previously evaluated 4D flow analysis method, which has shown high intra- and inter-observer reproducibility, was used for the analysis and quantification of the LV flow components [31]. The LV end-diastolic (EDV) and end-systolic (ESV) volumes were segmented, following the endocardial contour of the compact ventricular myocardium, from the short-axis image stack guided by long-axis images, using research segmentation software (Segment, Medviso AB, Lund, Sweden). End-diastolic (ED) and end-systolic (ES) timeframes were defined using visual observation in two-, three- and four-chamber images as the first time frame in which the atrioventricular valves were completely closed and the last time frame in which the atrioventricular valves were completely closed, respectively. Visual inspection of the aortic valve was also performed to confirm that it was closed at both ES and ED. The segmentation was superimposed on the magnitude data of the 4D flow CMR in order to manually correct potential mismatches due to patient motion in the short and long axis orientation.

From the segmented EDV, a pathline was emitted from the center of each voxel at ED and traced forwards and backwards to the time of ES, thus encompassing one cardiac cycle. Each pathline is considered to represent a volume of blood corresponding to the voxel size. At end-systole, the segmented ESV was used to determine the origin (backwards traces) and destination (forwards traces) of each pathline.

Speed plots extracted from the 4D flow data at the vicinity of the mitral valve and the aortic valve orifices, respectively, were used together with 4D flow data visualizations to determine time of ED and ES in order to separate the cardiac cycle into systole and diastole. Diastole was then further divided into early (E-wave) and late (A-wave) filling, which were defined as the interval from onset diastole until mid-diastasis and the interval from mid-diastasis until ED, respectively. The time of mid-diastasis was defined visually as the time frame at which the lowest number of pathlines crossed the mitral valve plane.

**Fig. 2:**
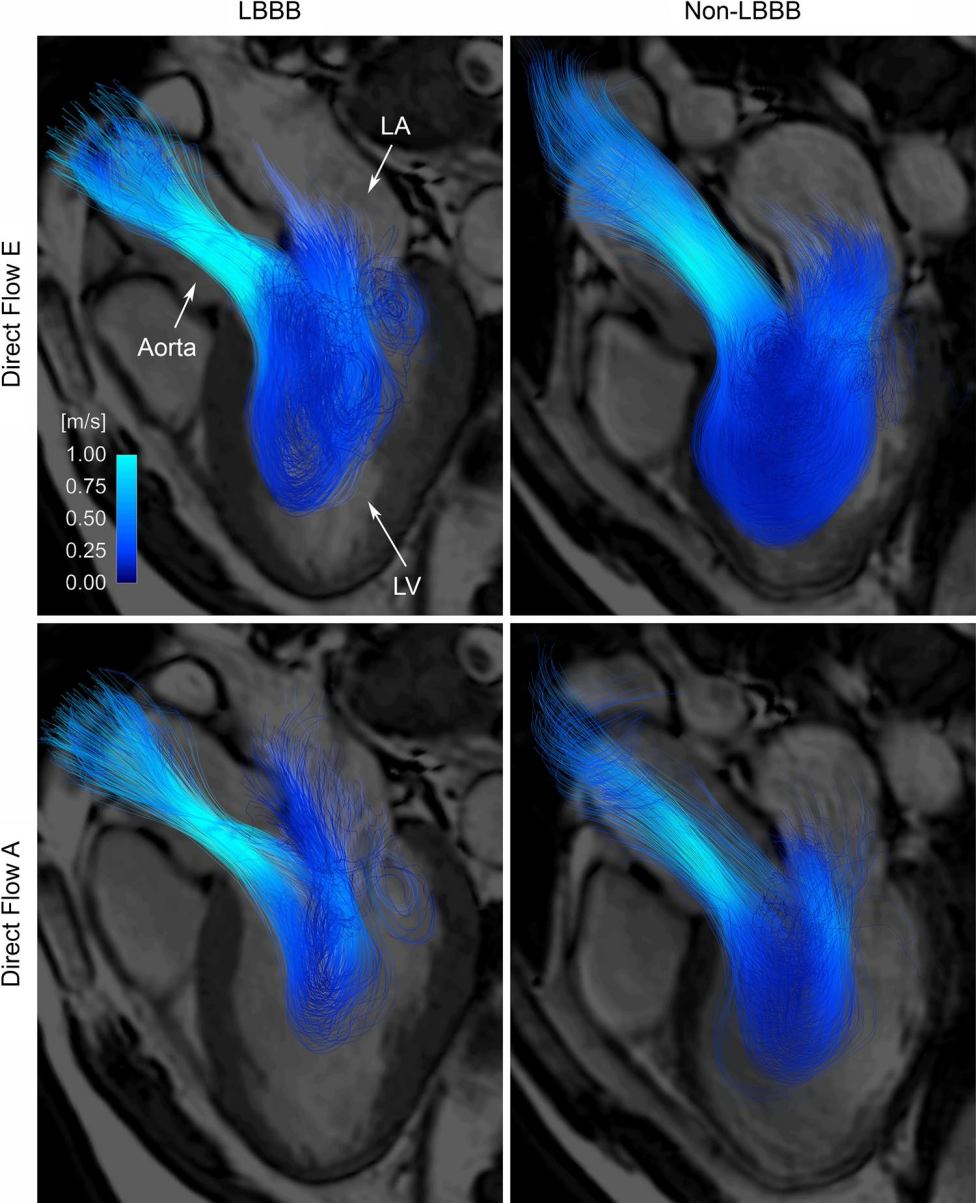
Pathlines through the LV for the *Direct Flow* component entering during early diastolic filling (E, top panels) and late diastolic filling (A, bottom panels) in a LBBB patient (left panels) and matched patient (right panels), colored according to speed. *LA* left atrium, *LV* left ventricle

The blood volume was divided into four functional flow components [13]: *Direct Flow*: blood that enters the LV during diastole and leaves the LV during systole in the analyzed heart beat; *Retained Inflow*: blood that enters the LV during diastole but does not leave during systole in the analyzed heart beat; *Delayed Ejection Flow*: blood that resides inside the LV during diastole and leaves during systole in the analyzed heart beat; and *Residual Volume*: blood that resides within the LV for at least two cardiac cycles. Additionally, four flow components were created as subvolumes of the inflow components according to the two diastolic phases: *Direct Flow E* and *Direct Flow A: Direct Flow* entering the LV during E-wave and A-wave respectively; and *Retained Inflow E* and *Retained Inflow A: Retained Inflow* entering during E-wave and A-wave respectively. The LV pathlines were visualized over time by using a commercially available flow visualization software (EnSight, CEI, Apex, NC) (Fig. 2, Supplementary Video 1 and 2).

For each flow component (eight in total) the kinetic energy (KE) was calculated over the entire cardiac cycle as the sum of KE for all pathlines comprising each component using the volume represented by each trace, its velocity and the density of blood. Each component’s KE was also normalized to its volume (KE/ml). The KE and KE/ml at the time of ED was used for comparison and considered as pre-systolic energetic values.

All data sets went through a careful quality control which included visual inspection of emitted pathlines to identify any aberrancy related to poor image quality from noise or movement, and a comparison between the ventricular inflow and outflow volumes. If the inflow-outflow discrepancy was > 15%, patient data were excluded from further analysis.

### Statistical evaluation

The Kolmogorov–Smirnov test was used to check the data for normal distribution. All quantitative parameters except four (volume for *Direct Flow E* and *Residual Volume*, KE at ED for *Direct Flow A* and KE/ml at ED for *Residual Volume*) showed a normal distribution. Inter-group comparisons were analyzed using a Student’s *t* test for unpaired observations for normally distributed data and Mann–Whitney U test for non-normally distributed data as well as for the comparison of NYHA classification between the two groups. All values are given as group means ± 1SD unless otherwise specified. Linear regression was used to analyze the association between quantitative parameters and QRS duration. The statistical significance was set to P < 0.05. The software used for the statistical analyses was Statistica 9.1 (StatSoft, Tulsa, OK).

## Results

As shown in Table 1, there was no intergroup difference in age, gender, body surface area, heart rate, blood pressure, etiology of heart failure, NYHA classification, first-degree atrioventricular block, LV diastolic function, LVEF or LVEDV-index, whereas, as expected, the QRS duration was significantly longer in patients with LBBB (P < 0.001) (Table 1). The regional LV myocardial function for each LBBB patient with ICM and respective matched patient were comparable. As an indicator of data quality, LV inflow and outflow volume were compared; the mean for all patients was 74 ± 16 ml and 70 ± 15 ml, respectively.

**Table 1:** Demographic and clinical parameters

	LBBB group	Non-LBBB group	P value
Age (years)	61 ± 14	58 ± 16	0.676
Gender (female:male)	2:9	2:9	–
Height (cm)	173 ± 9	172 ± 7	0.763
Weight (kg)	88 ± 20	85 ± 11	0.688
BSA (m2)	2.0 ± 0.2	2.0 ± 0.2	0.728
Heart rate (bpm)	71 ± 13	68 ± 9	0.595
BP systolic (mmHg)	135 ± 15	132 ± 21	0.733
BP diastolic (mmHg)	76 ± 6	82 ± 17	0.357
Etiology (ICM:NICM)	4:7	4:7	–
NYHA classification	2 (1–3)	2 (1–3)	0.401
QRS duration (ms)	160 ± 20	106 ± 9	< 0.001
AV block I (number of patients)	1	1	–
LV diastolic function according to echo Doppler indices			
Normal	6	6	–
Relaxation abnormality	4	4	–
Pseudonormal filling	1	1	–
Restrictive filling	0	0	–
MRI data			
LVEF (%)	34 ± 9	37 ± 9	0.550
LVEDV (ml)	261 ± 90	235 ± 72	0.462
LVEDV-index (ml/m^2^)	129 ± 48	116 ± 32	0.455
LVESV (ml)	177 ± 82	154 ± 69	0.490
Medication (number of patients)			
Beta-blocker	11	8	–
ACE-I or ARB	11	9	–
Calcium channel blocker	2	0	–
Nitrate (long lasting)	0	1	–
Antithrombotic/anticoagulant	6	8	–
Statin	6	7	–
Diuretics	6	4	–

Mean ± SD, except for NYHA classification: median (range). *ACE-I* angiotensin converting enzyme inhibitor, *ARB* angiotensin receptor blocker, *AV* atrioventricular, *BP* blood pressure, *bpm* beats per minute, *BSA* body surface area, *NICM* nonischemic cardiomyopathy, *EDV* end-diastolic volume, *ESV* end-systolic volume, *EF* ejection fraction, *ICM* ischemic cardiomyopathy, *LV* left ventricle, *NYHA* New York Heart Association. The echo Doppler indices for LV diastolic function was applied according to standard recommendations [29]

**Fig. 3:**
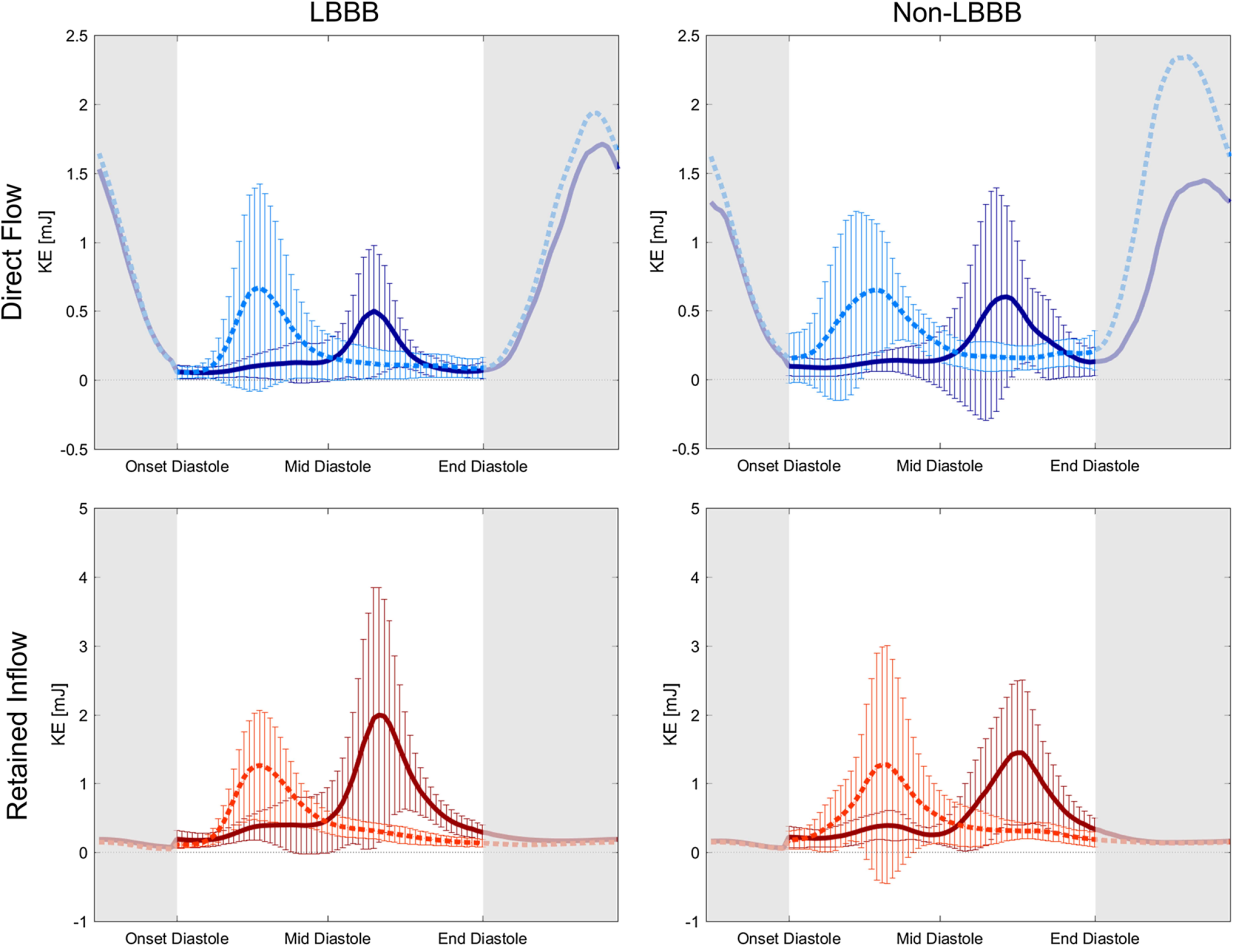
Kinetic energy (mJ) over diastole (white area; mean ± SD) and systole (grey areas; mean) for *Direct Flow* (light and dark blue; top panels) and *Retained Inflow* (light and dark red; bottom panels) in LBBB-patients (left panels) and matched patients (right panels), colored according to diastolic phase: early diastolic inflow in light color (dotted line) and late diastolic phase in dark color (solid line). Note the larger KE at end-diastole for the early diastolic *Direct Flow* in non-LBBB patients compared to that of LBBB patients. *KE* kinetic energy, *mJ* millijoule

All subjects showed a LV inflow velocity peak at both early and late diastolic filling with the exception of two patients with LBBB that only showed a single diastolic peak due to superimposition of E and A peaks; these patients and their corresponding matched patients were not included in the early vs late diastolic filling comparisons. For the remaining nine LBBB patients and nine matched patients there was no difference in the aforementioned demographic and clinical parameters. These eighteen patients presented an early and late diastolic peak in KE for both the *Direct Flow* and *Retained Inflow* components (Fig. 3).

**Fig. 4:**
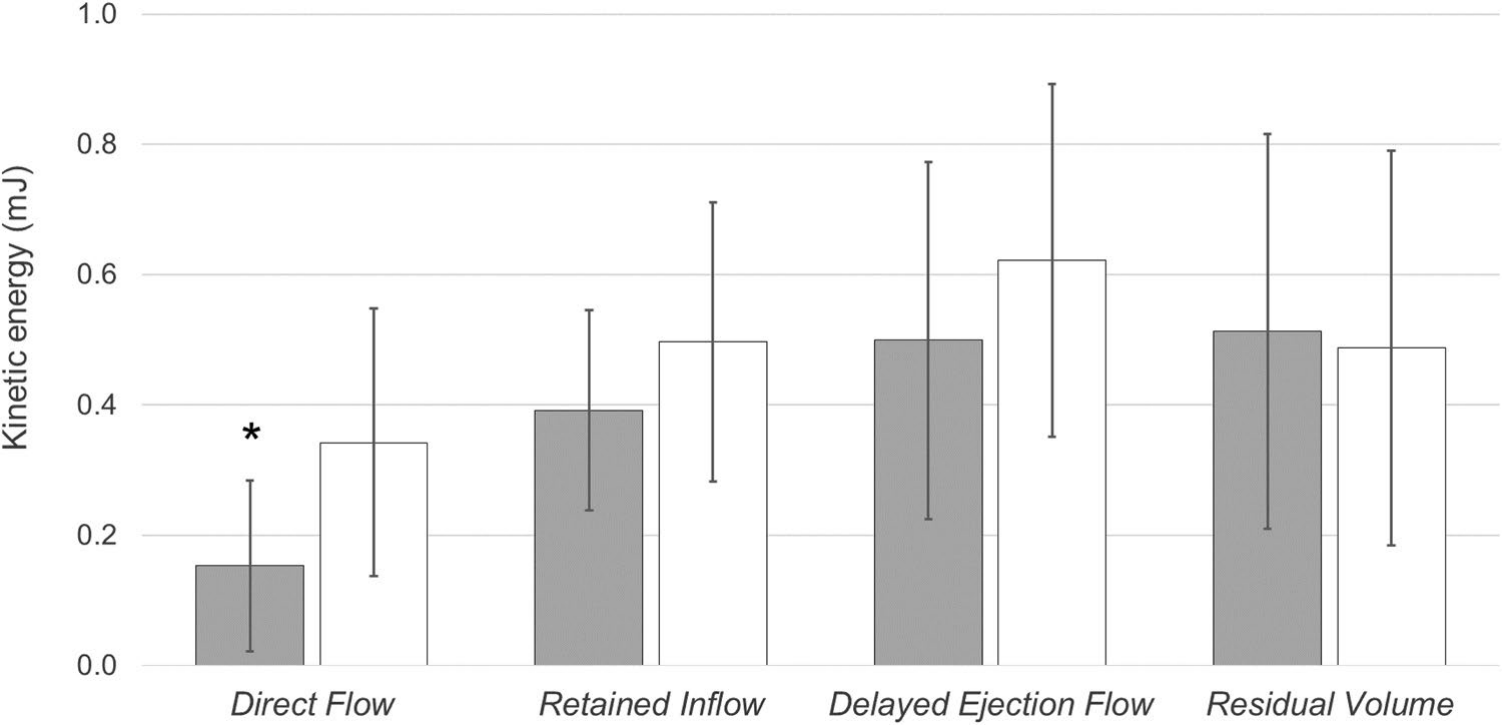
Kinetic energy (mJ) at end-diastole for *Direct Flow, Retained Inflow, Delayed Ejection Flow* and *Residual Volume* in LBBB patients (grey) and matched patients without LBBB (white). *mJ* millijoule. *P = 0.018 versus *Direct Flow* in patients without LBBB

**Fig. 5:**
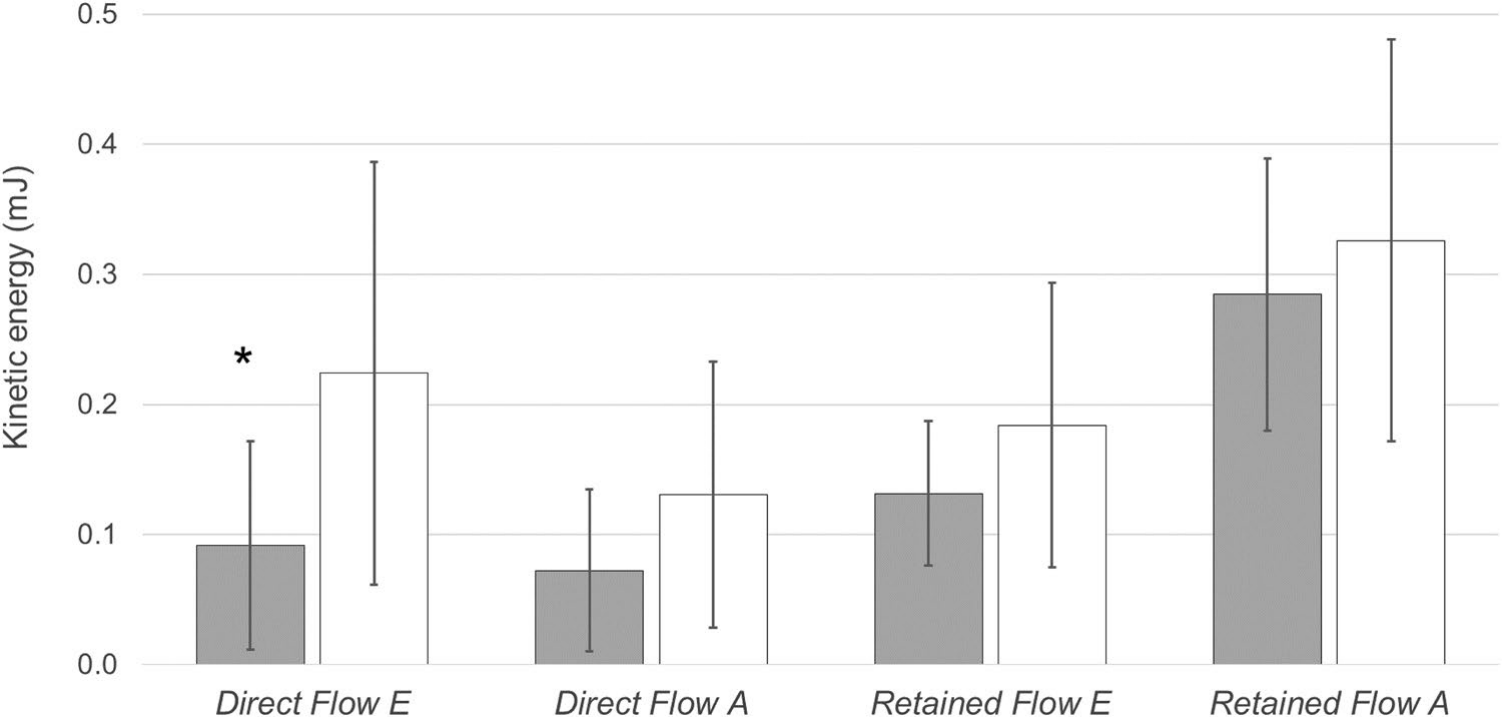
Kinetic energy (mJ) at end-diastole for *Direct Flow E, Direct Flow A, Retained Inflow E* and *Retained Inflow A* in LBBB patients (grey) and matched patients without LBBB (white). *mJ* millijoule. *P = 0.043 versus *Direct Flow E* in patients without LBBB

The volume of each flow component was not significantly different between the two groups (Table 2). The *Direct Flow* KE at ED was lower in patients with LBBB compared to patients without LBBB (P = 0.018) (Fig. 4). Furthermore the KE at ED for the *Direct Flow E* component was also lower in LBBB patient compared to matched patients (P = 0.043) (Fig. 5). However, there was no difference in KE at ED for *Direct Flow A* and the remaining components between the two groups (Figs. 4, 5).

**Table 2:** 4D flow CMR data in patients with and without LBBB

	LBBB group	Non-LBBB group	P value
Volume (ml)			
Direct Flow	18.9 ± 10.5	26.2 ± 12.4	0.148
Direct Flow E	9.0 ± 5.6	14.4 ± 9.0	0.094
Direct Flow A	10.4 ± 4.8	12.5 ± 5.8	0.416
Retained Inflow	53.9 ± 15.8	49.2 ± 15.9	0.498
Retained Inflow E	24.1 ± 9.7	23.3 ± 11.2	0.882
Retained Inflow A	33.1 ± 8.1	27.8 ± 8.8	0.208
Delayed Ejection Flow	48.9 ± 15.3	46.5 ± 11.5	0.675
Residual Volume	119.7 ± 73.7	99.0 ± 62.8	0.478
KE/volume at ED (µJ/ml)			
Direct Flow	7.2 ± 3.3	12.0 ± 4.3	0.008
Direct Flow E	8.7 ± 5.2	15.3 ± 5.5	0.017
Direct Flow A	6.1 ± 2.8	9.0 ± 4.6	0.116
Retained Inflow	7.1 ± 1.4	10.0 ± 3.5	0.018
Retained Inflow E	5.5 ± 0.8	8.3 ± 3.4	0.030
Retained Inflow A	8.5 ± 1.6	11.6 ± 4.5	0.075
Delayed Ejection Flow	10.1 ± 5.1	13.6 ± 5.0	0.125
Residual Volume	4.3 ± 0.7	5.0 ± 2.6	0.847

Mean ± SD. *ED* end-diastole, *KE* kinetic energy

When normalizing the KE for each component to its volume, KE/ml at ED for *Direct Flow, Direct Flow E, Retained Inflow* and *Retained Inflow E* components was lower in patients with LBBB compared to matched patients (Table 2). No intergroup difference in KE/ml at ED was found for *Direct Flow A*, *Retained Inflow A* and the remaining components (Table 2).

KE at ED of *Direct Flow* and *Direct Flow E* related weakly to moderately to QRS duration (Table 3), whereas there was no relation between KE at ED of *Direct Flow A* and QRS duration. KE/ml at ED of *Direct Flow* and *Direct Flow E* related moderately to QRS duration; no relation was found between KE/ml at ED of *Direct Flow A* and QRS duration.

**Table 3:** Regression analysis of Direct Flow parameters versus QRS-duration

	R^2^ value	P value
Volume (ml)		
Direct Flow	0.089	0.178
Direct Flow E	0.190	0.070
Direct Flow A	0.034	0.467
KE at ED (µJ)		
Direct Flow	0.234	0.022
Direct Flow E	0.249	0.035
Direct Flow A	0.110	0.178
KE/volume at ED (µJ/ml)		
Direct Flow	0.329	0.005
Direct Flow E	0.317	0.015
Direct Flow A	0.187	0.073

*ED* end-diastole, *KE* kinetic energy

## Discussion

In this study LV 4D flow patterns and energetics were investigated in heart failure patients with LBBB and compared to matched patients without LBBB. Although the volume and subvolumes of the *Direct Flow* were equal between the groups, the pre-systolic kinetic energy of the *Direct Flow*, and of the portion of *Direct Flow* entering during early but not late diastolic filling, were reduced in LBBB patients compared to matched patients with normal conduction.

In a recent 4D flow CMR study, the *Direct Flow* proportion of the LVEDV was lower in heart failure patients with mild to moderate LV remodeling and dysfunction compared to healthy controls (20 vs. 38%) [19]. Also, the *Direct Flow* KE at ED was lower in these heart failure patients compared to healthy subjects, but the reduction in KE was secondary to lower *Direct Flow* volume. In the current study in heart failure patients with at least moderate LV remodeling and dysfunction, the *Direct Flow* proportion of the LVEDV was even lower (approximately 12%). In contrast to the earlier study, in this study we found that the lower *Direct Flow* KE at ED in LBBB patients compared to the matched patients was not attributable to lower *Direct Flow* volume. Therefore, the difference was due to lower velocity of the blood of the *Direct Flow* component at ED, and in particular, of the *Direct Flow* entering during early diastolic filling.

A consequence of the dyssynchronous electromechanical function of LBBB hearts is that the early diastolic relaxation of the LV myocardium is delayed compared to the right ventricular myocardium, and typically the outward motion of the LV lateral wall is delayed relative to other ventricular walls. Moreover, as the diastolic filling phase is shorter in LBBB hearts compared to hearts with normal conduction, the timing between early and late diastolic inflow is compromised. Previous studies in normal hearts [13, 18] have shown that *Direct Flow* entering during early diastolic filling proceeds deeper into the LV chamber, compared to the *Direct Flow* entering during late filling, before it turns and is directed towards the LVOT. These studies have also demonstrated that the atrial contraction contributes to an increase in the rotational speed of the anteroseptal part of the submitral inflow vortex and accelerates blood flow towards the LVOT. This “boost” in late diastolic blood velocity can contribute to some increase in velocity of the *Direct Flow* that entered during early filling (Fig. 3, right upper panel). In LBBB hearts compared to hearts with normal conduction, the delayed entering of the DF during early filling could hamper its positioning close to the LV outflow region at onset of atrial contraction, which in turns makes it less influenced by the “boost” in late diastolic velocity (Fig. 3, left upper panel). This mechanism may contribute to the lower KE at ED of *Direct Flow* entering during early diastolic filling in LBBB patients compared to matched patients.

KE/ml at ED of *Direct Flow* and early inflowing *Direct Flow* were found to relate moderately to QRS duration. Although differences in QRS duration can only explain approximately 30% of these 4D flow parameters, they suggest that preservation of KE at ED for early inflowing *Direct Flow* diminishes with greater conduction abnormality.

Recently, Gurel and co-workers studied LV flow in patients with LBBB using echocardiographic particle image velocimetry [32]. They found that LBBB patients, compared to healthy subjects, had a shorter diastolic filling phase, delayed vortex formation during early diastolic filling and diminished vortex KE preservation. These 2D echo-PIV findings are in line with the current 3D CMR results in terms of altered energetics of LV inflow, secondary to delayed early diastolic relaxation, implying impaired LV diastolic function. However, in the current study, KE for the whole end-diastolic blood volume is computed throughout the cardiac cycle, including the KE possessed by inflow vortices.

Also, in an elegant echo-PIV study in eleven heart failure patients with implanted CRT, Goliasch and co-workers showed that the onset of the LV filling vortex formation during early diastole was significantly delayed when the CRT was deactivated and was immediately reversed upon reactivation [11]. The authors stated that this delay was due to a disorganized LV inflow with reduced acceleration during early diastole caused by LV dyssynchrony. Moreover, CRT deactivation prolonged the isovolumic contraction phase, which closely correlated with the delay in early diastolic vortex formation. These interesting 2D flow findings, although in line with the present 3D flow findings, cannot be directly compared as deactivation of CRT for a short moment probably does not entirely reflect the patients underlying LV mechanical dyssynchrony patterns.

On the contrary to the early diastolic filling, the late diastolic inflow is dependent on atrial contraction and thus less influenced by the dyssynchronous LV relaxation and motion, which could explain the absence of any intergroup differences at late diastole.

Stratification according to etiology of heart failure (ICM and NICM) was done. KE/ml at ED of *Direct Flow* was significantly lower in NICM patients with LBBB compared to NICM patients without LBBB. Such difference was not observed between the ICM patient groups, however, only four patients were included in each group. These interesting very preliminary findings need to be further investigated in future larger cohort studies.

Quantification of ventricular inflow with respect to the continuously changing position of the AV valve has previously been accurately assessed using retrospective valve tracking [16]. Implementation of this technique in the current 4D flow analysis method may contribute to additional information related to the complex nature of mitral inflow in patients with LBBB.

The present findings suggest that 4D flow specific measures could serve as markers of LV mechanical dyssynchrony in heart failure patients. A reduced KE preservation at ED of LV inflow passing directly to outflow could lead to elevated filling pressures. Moreover, as the *Direct Flow* is destined for ejection during the subsequent systole, a lower KE at pre-systole in LBBB hearts could indicate that incremental contribution from systolic contraction will be required for its ejection. Accordingly, these flow-specific markers may reflect incremental impairment in diastolic function that could contribute to progressive adverse cardiac remodeling and possibly be investigated as predictors of response to CRT.

### Limitations

In this unique CMR study, using advanced methodology, the findings relate to a relatively small number of heart failure patients. However, thorough work was put into matching the two heart failure patient groups to each other; for every patient with LBBB included, another patient without LBBB but with the same age, gender and heart rate as well as with similarly dysfunctional and remodeled LV was identified. Future studies with larger patient cohorts are required to validate current findings; e.g. for comparison according to etiology of heart failure and to assess the benefit in predicting response to CRT.

Ischemic heart disease related regional LV wall motion abnormality may influence intraventricular flow dynamics, regardless of the presence of LBBB. The present study included four LBBB patients and four matched non-LBBB patients with ICM. The pattern of regional LV wall motion abnormality in these patients were assessed in order to assure that each LBBB patient with ICM were matched to another ICM patient without LBBB but with similar pattern of regional LV myocardial function.

Two included ICM patient with LBBB had previously undergone coronary artery bypass grafting. As patients that have undergone such open heart surgery may demonstrate postoperative paradoxical septal motion, one LBBB patient was matched to a patient who had also had previous coronary artery bypass grafting.

The current data relate to subjects in the supine position at rest and in sinus rhythm. All CMR data were acquired during end-expiration and are only representative for the end-expiratory volume and pressure situation.

## Conclusions

4D flow patterns and energetics in myopathic LVs with and without LBBB demonstrate reduced pre-systolic KE of *Direct Flow* in patients with LBBB compared to matched patients with normal conduction. This reduction is attributed to *Direct Flow* entering during early diastole, and may reflect incremental impairment of early diastolic function and subsequent impairment of efficient systolic ejection related to dyssynchrony in these failing ventricles. These intriguing findings propose that 4D flow specific measures can serve as markers of LV mechanical dyssynchrony in heart failure patients, and could possibly be investigated as predictors of response to CRT.

## Electronic supplementary material

Below is the link to the electronic supplementary material.

Supplementary material 1—Pathline visualization of the *Direct Flow* entering the left ventricle (LV) during early diastolic filling (E, blue to turquoise) and late diastolic filling (A, brown to yellow) in a LBBB patient. Pathlines are color coded according to speed. A semi-transparent three-chamber images provide anatomical orientation (MP4 2863 KB)

Supplementary material 2—Pathline visualization of the *Direct Flow* entering the left ventricle (LV) during early diastolic filling (E, blue to turquoise) and late diastolic filling (A, brown to yellow) in a matched patient without LBBB. Pathlines are color coded according to speed. A semi-transparent three-chamber images provide anatomical orientation (MP4 3223 KB)

## References

[1] Kashani A, Barold SS (2005). Significance of QRS complex duration in patients with heart failure. J Am Coll Cardiol.

[2] Xiao HB, Roy C, Fujimoto S, Gibson DG (1996). Natural history of abnormal conduction and its relation to prognosis in patients with dilated cardiomyopathy. Int J Cardiol.

[3] Grines CL, Bashore TM, Boudoulas H, Olson S, Shafer P, Wooley CF (1989). Functional abnormalities in isolated left bundle branch block. The effect of interventricular asynchrony. Circulation.

[4] Witt CM, Wu G, Yang D, Hodge DO, Roger V, Cha YM (2016). Outcomes with left bundle branch block and mildly to moderately reduced left ventricular function. JACC Heart Fail.

[5] Mann DL (2004). Basic mechanisms of left ventricular remodeling: the contribution of wall stress. J Card Fail.

[6] Abraham WT, Fisher WG, Smith AL, Delurgio DB, Leon AR, Loh E, Kocovic DZ, Packer M, Clavell AL, Hayes DL, Ellestad M, Trupp RJ, Underwood J, Pickering F, Truex C, McAtee P, Messenger J (2002). Cardiac resynchronization in chronic heart failure. N Engl J Med.

[7] Lecoq G, Leclercq C, Leray E, Crocq C, Alonso C, de Place C, Mabo P, Daubert C (2005). Clinical and electrocardiographic predictors of a positive response to cardiac resynchronization therapy in advanced heart failure. Eur Heart J.

[8] Sengupta PP, Pedrizzetti G, Kilner PJ, Kheradvar A, Ebbers T, Tonti G, Fraser AG, Narula J (2012). Emerging trends in CV flow visualization. J Am Coll Cardiol Imaging.

[9] Kilner PJ, Yang GZ, Wilkes AJ, Mohiaddin RH, Firmin DN, Yacoub MH (2000). Asymmetric redirection of flow through the heart. Nature.

[10] Kim WY, Walker PG, Pedersen EM, Poulsen JK, Oyre S, Houlind K, Yoganathan AP (1995). Left ventricular blood flow patterns in normal subjects: a quantitative analysis by three-dimensional magnetic resonance velocity mapping. J Am Coll Cardiol.

[11] Goliasch G, Goscinska-Bis K, Caracciolo G, Nakabo A, Smolka G, Pedrizzetti G, Narula J, Sengupta PP (2013). CRT improves LV filling dynamics: insights from echocardiographic particle imaging velocimetry. JACC Cardiovasc Imaging.

[12] Dyverfeldt P, Bissell M, Barker AJ, Bolger AF, Carlhäll CJ, Ebbers T, Francios CJ, Frydrychowicz A, Geiger J, Giese D, Hope MD, Kilner PJ, Kozerke S, Myerson S, Neubauer S, Wieben O, Markl M (2015). 4D flow cardiovascular magnetic resonance consensus statement. J Cardiovasc Magn Reson.

[13] Bolger AF, Heiberg E, Karlsson M, Wigström L, Engvall J, Sigfridsson A, Ebbers T, Kvitting JP, Carlhäll CJ, Wranne B (2007). Transit of blood flow through the human left ventricle mapped by cardiovascular magnetic resonance. J Cardiovasc Magn Reson.

[14] Carlhäll CJ, Bolger AF (2010). Passing strange: flow in the failing ventricle. Circ Heart Fail.

[15] Carlsson M, Heiberg E, Toger J, Arheden H (2012). Quantification of left and right ventricular kinetic energy using four-dimensional intracardiac magnetic resonance imaging flow measurements. Am J Physiol Heart Circ Physiol.

[16] Westenberg JJ, Roes SD, Marsan NA, Binnendijk NM, Doornbos J, Bax JJ, Reiber JH, de Roos A, van der Geest RJ (2008). Mitral valve and tricuspid valve blood flow: accurate quantification with 3D velocity-encoded MR imaging with retrospective valve tracking. Radiology.

[17] Markl M, Carr M, Ng J, Lee D, Jarvis K, Carr J, Goldberger J (2016). Assessment of left and right atrial 3D hemodynamics in patients with atrial fibrillation: a 4D flow MRI study. Int J Cardiovasc Imaging.

[18] Eriksson J, Dyverfeldt P, Engvall J, Bolger AF, Ebbers T, Carlhäll CJ (2011). Quantification of presystolic blood flow organization and energetics in the human left ventricle. Am J Physiol Heart Circ Physiol.

[19] Eriksson J, Bolger AF, Ebbers T, Carlhäll CJ (2013). Four-dimensional blood flow-specific markers of LV dysfunction in dilated cardiomyopathy. Eur Heart J Cardiovasc Imaging.

[20] Svalbring E, Fredriksson A, Eriksson J, Dyverfeldt P, Ebbers T, Bolger AF, Engvall J, Carlhäll CJ (2016). Altered diastolic flow patterns and kinetic energy in subtle left ventricular remodeling and dysfunction detected by 4D flow MRI. PLoS ONE.

[21] Yu CM, Zhang Q, Fung JW, Chan HC, Chan YS, Yip GW, Kong SL, Lin H, Zhang Y, Sanderson JE (2005). A novel tool to assess systolic asynchrony and identify responders of cardiac resynchronization therapy by tissue synchronization imaging. J Am Coll Cardiol.

[22] Yu CM, Gorcsan J, Bleeker GB, Zhang Q, Schalij MJ, Suffoletto MS, Fung JW, Schwartzman D, Chan YS, Tanabe M, Bax JJ (2007). Usefulness of tissue Doppler velocity and strain dyssynchrony for predicting left ventricular reverse remodeling response after cardiac resynchronization therapy. Am J Cardiol.

[23] Buss SJ, Humpert PM, Bekeredjian R, Hardt SE, Zugck C, Schellberg S, Bauer A, Filusch A, Kuecherer H, Katus HA, Korosoglou G (2009). Echocardiographic phase imaging to predict reverse remodeling after cardiac resynchronization therapy. J Am Coll Cardiol Imaging.

[24] Han Y, Chan J, Haber I, Peters DC, Zimetbaum PJ, Manning WJ, Yeon SB (2010). Circumferential myocardial strain in cardiomyopathy with and without left bundle branch block. J Cardiovasc Magn Reson.

[25] Rosen BD, Lardo AC, Berger RD (2006). Imaging of myocardial dyssynchrony in congestive heart failure. Heart Fail Rev.

[26] Brunekreeft JA, Graauw M, de Milliano PAR, Keijer JT (2007). Influence of left bundle branch block on left ventricular volumes, ejection fraction and regional wall motion. Neth Heart J.

[27] Xiang QS (1995). Temporal phase unwrapping for CINE velocity imaging. J Magn Reson Imaging.

[28] Surawicz B, Childers R, Deal BJ, Gettes LS, Bailey JJ, Gorgels A, Hancock EW, Josephson M, Kligfield P (2009). AHA/ACCF/HRS recommendations for the standardization and interpretation of the electrocardiogram: part III: intraventricular conduction disturbances a scientific statement from the American Heart Association Electrocardiography and Arrhythmias Committee, Council on Clinical Cardiology; the American College of Cardiology Foundation; and the Heart Rhythm Society endorsed by the International Society for Computerized Electrocardiology. Circulation.

[29] Nagueh S, Smiseth O, Appleton C, Byrd BR,  Dokainish H, Edvardsen T, Flachskampf F, Gillebert T, Klein A, Lancellotti P, Marino P, Oh J, Alexandru Popescu B, Waggoner A (2016). Recommendations for the evaluation of left ventricular diastolic function by echocardiography: an update from the American Society of Echocardiography and the European Association of Cardiovascular Imaging. Eur Heart J Cardiovasc Imaging.

[30] Nishimura RA, Otto CM, Bonow RO, Carabello BA, Erwin JP, Guyton RA, O’Gara PT, Ruiz CE, Skubas NJ, Sorajja P, Sundt TM, Thomas JD (2014). 2014 AHA/ACC guideline for the management of patients with valvular heart disease: a report of the American College of Cardiology/American Heart Association Task Force on Practice Guidelines. Circulation.

[31] Eriksson J, Carlhäll CJ, Dyverfeldt P, Engvall J, Bolger AF, Ebbers T (2010). Semi-automatic quantification of 4D left ventricular blood flow. J Cardiovasc Magn Reson.

[32] Gürel E, Prinz C, Van Casteren L, Gao H, Willems R, Voigt JU (2016). The impact of function-flow interaction on left ventricular efficiency in patients with conduction abnormalities: a particle image velocimetry and tissue Doppler study. J Am Soc Echocardiogr.

